# Educating patients about patient-reported outcomes—are we there yet?

**DOI:** 10.1186/s41687-024-00745-5

**Published:** 2024-09-30

**Authors:** Elizabeth Unni, Maud M. van Muilekom, Kate Absolom, Bishnu Bajgain, Lotte Haverman, Maria Santana

**Affiliations:** 1 Social, Behavioral, and Administrative Pharmacy, Touro College of Pharmacy, New York, NY USA; 2 Emma Children’s Hospital, Child and Adolescent Psychiatry & Psychosocial Care, Amsterdam UMC Location University of Amsterdam, Amsterdam, The Netherlands; 3 Amsterdam Reproduction & Development Research Institute, Child Development, Amsterdam UMC, Amsterdam, The Netherlands; 4 Amsterdam Public Health Research Institute, Mental Health and Personalized Medicine, University of Leeds, Amsterdam, The Netherlands; 5 Leeds Institute of Health Services Research, Leeds Institute of Medical Research, University of Leeds, Leeds, UK; 6 Department of Community Health Sciences, University of Calgary, Calgary, AB Canada; 7 Amsterdam Public Health Institute, Mental Health and Digital Health, Amsterdam, The Netherlands; 8 Patient and Family-Centered Care Scientist, Departments of Pediatrics and Community Health Sciences, Cumming School of Medicine, University of Calgary, Alberta, Canada

**Keywords:** Patient reported outcome measures, Clinical practice, Patient education

## Abstract

**Background:**

Using Patient Reported Outcome Measures (PROMs) in clinical settings can improve patient outcomes by enhancing communication between patient and provider. There has been significant improvements in the development of PROMs, their implementation in routine patient clinical care, training physicians and other healthcare providers to interpret the PROMs results to identify any issues reported by the patient, and to use the PROMs results to provide or modify the treatment.

**Main body:**

Despite the increased use of PROMs, the lack of PROM completion by patients is a major concern in the optimal use of PROMs. Studies have shown several reasons why patients do not complete PROMs and one of the reasons is their lack of understanding of the significance of PROMs and their utility in their clinical care. While examining the various strategies that can be used to improve the uptake of PROM completion by patients, educating patients about the use of PROMs has been recommended. There is less evidence on how patients are trained or educated about PROMs. It may also be possible that the patient education strategies are not reported in the publications. This brings up the question of evaluation of the educational strategies used.

**Conclusion:**

Our symposium at the 2023 ISOQOL conference brought together a range of experiences and learning around patient-centered PROMs educational activities used in the Netherlands, Canada, and the UK. This commentary is aimed to describe the lay of the land about educational activities around the use of PROMs in clinical care for patients, recognizing the gaps, and posing questions to be considered by the research and clinical community.

## Background

The authors presented a symposium during the ISOQOL 2023 annual meeting which highlighted the need for improved reporting and guidance for supporting best practices in patient education on the use of PROMs in routine clinical care. This commentary is a summary of the symposium we presented.

Patient Reported Outcomes (PROs) is a status report of a patient’s health condition elicited directly from the patient and allows the patient’s values and perspectives to be reported without any interpretation of this response by a healthcare practitioner or anyone else. Patient-Reported Outcomes Measures (PROMs) are standardized and validated questionnaires (either disease-specific or general), administered on paper or electronically. Using PROMs in clinical care provides the data needed to support the co-design of care plans, enabling shared decision-making while providers adjust care and treatment in collaboration with patients [1–5]. A recent Cochrane systematic review as well as other studies have reported several benefits from using PROMs in routine clinical care including improvements in care planning and design for the patient, especially with physician-patient communication [5–7]. Though with less certainty, there is still evidence on how PROMs can improve diagnosis and recognition of clinical problems [5–7]. Compared to the objective clinical measures used by health care providers such as vital signs and lab values, PROMs when used effectively can empower patients and improve their experiences with the care provided to them. Measurement science has refined the development of PROMs to tailor their use to clinical care [8–9].

Central to the optimal use of PROMs by healthcare providers, is their completion by the patients, an often-encountered problem. Unlike clinical trials where patients are recruited for a study and often incentives are given to complete the PROMs, the completion of PROMs in a routine clinical setting can be a daunting task. Previous studies have reported the rate of PROM completion ranging from 50 to 80% in clinical care [10–12]. Though physicians and other healthcare providers are trained to interpret the PROMs results to identify any issues reported by the patient, to discuss them with the patients, and to provide or modify the treatment accordingly [13], there is a paucity of data in the literature about educating or training patients about PROMs, its utility in their clinical care, and how to effectively use the PROM reports in shared decision making with physicians.

In this commentary we will describe barriers to completing PROMs due to the lack of education for patients about the PROMs, we will provide available evidence on strategies to encourage patients to complete PROMs, and examples from specific clinics from the Netherlands, Canada and the UK.

## Main text

### Barriers to completing PROMs by patients

Some of the major barriers to the incompletion of PROMs by patients in clinical settings is patient’s inability (due to factors such as difficulty in reading, responding, recalling, and physical illness) to complete the PROMs, their lack of understanding about the goal of the PROM, and perceived irrelevance and lack of value [14]. Carfora et al. in their systematic review and meta-analysis of the patient’s experiences and perspectives of PROMs in clinical care reported the lack of relevance of individual PROM questions, not knowing the purpose of PROMs, patient’s perception that the use of PROMs is limited to clinician and/or research applications, and not understanding how the PROMs are utilized in their care [15]. In essence, the patients do not understand or perceive the value of PROMs in their treatment plans. A qualitative analysis by Aiyegbusi et al. of the use of PROMs in rare diseases demonstrated a lack of awareness in patients about PROMs in addition to literacy issues [16]. The issue of patients, especially from diverse and underrepresented communities, needing help in completing surveys due to low education was brought up by Hyland et al. in their review [17].

### Strategies to improve PROM completion by patients

While healthcare organizations offered training programs to clinicians to understand the value, utility, and interpretation of PROMs [12], less attention has been given to educating patients and their caregivers about the use and value of PROMs in their clinical care. Some of the current strategies used to increase the response rate to PROMs are text messaging, the Plan-Do-Study-Act (PDSA) approach for implementing PROMs, utilizing the interoperability of PROMs within electronic health records to tailor patient care, and using a web-mail-phone approach to encourage PROM completion [18–20]. Literature explains the involvement of patients when developing and choosing PROMs and even having PROM committees in the clinics. But the breadth of information shared on how patients are trained or educated in PROMs can be limited and often the focus is on the practical aspects (e.g. completing the PROMs such as logging into online systems, completing PROMs, or what to do about lost login details and passwords). However, what is lacking is educating the everyday patient in the clinic about the use and value of PROMs, including the value of each PROM item. Patients also need to be educated about the utility of PROMs as part of their care to help engagement, and empowerment while speaking with their healthcare providers and how patients are educated including when, where, and by whom. Anecdotally, clinicians and researchers do report some aspects of patient education within PROM implementation initiatives. It is possible that patients are educated about PROMs, but the patient education strategies are not reported in the publications.

A review that was undertaken by Yang et al. to understand the strategies that can be used to facilitate PROM uptake recommended providing comprehensive instructions to patients on how to complete PROMs, interpret PROMs, and discuss issues during clinical consults [21]. Hyland et al. also recommended using video tutorials for ePROM completion among elderly patients, especially those with low income and education [17]. Palos et al. reported educating patients on the use of PROMs using a multiprong approach including a one-page tip sheet to explain the role and value of PROMs, instructions on how to access and complete the PROMs in the patient portal, using the portal to send messages to patients to encourage them to complete the PROMs, and developing a video to educate the patients while they are waiting to be seen by the clinicians [22]. The clinical team did this patient education based on the recommendation from the PROM committee in the clinic, which recognized the need for educational materials and training needed to facilitate patient use and acceptance of PROMs.

Example from Netherlands: In Netherlands, where they have experience with implementing PROMs in clinical practice for over 15 years using the KLIK PROM portal (www.hetklikt.nu) and Epic (the most common electronic health record in e.g. the Netherlands and USA), they have implemented several educational activities for patients such as psycho-educational flyers and information letters for patients about the goal and importance of completing PROMs in clinical practice, instructional videos on how to complete PROMs and an informative PROM website with relevant links for patients [23]. Also, patients are engaged in selecting PROMs and developing optimal PROM visualizations. Finally, educational videos and a topic list are available to help patients discuss PROMs during the consultation, and a patient engagement game was developed to incorporate what matters to them in hospital care, research, and policy. However, data on PROM completion rates with the use of educational activities are not currently extracted at the KLIK PROM portal.

Example from Canada: In Canada, the Summit program at the University of Calgary, which is a new program, engages patients in preparing educational material for them and uses educational videos and flyers to educate patients about PROMs, their objectives, and the importance of completing them for their clinical care [24–25]. These educational videos and flyers are kept at the clinics and added at the first part of the e-PROMs completion process so that patients understand the goals and importance of completing the PROMs. This has resulted in the PROM completion rate at 70%.

Example from UK: In terms of best practices for the design, content, and delivery of patient-facing PROMs education and information materials little guidance is currently available. In projects previously delivered by the Patient-Centered Outcomes Research team, the University of Leeds in the UK, input from medical education experts was obtained to help create training materials using videos of simulated patient cancer consultations where PROMs data were used [12]. The approach applied principles from adult learning theory to help guide the development of interactive and experiential training. Similar approaches may be beneficial for similar patient-focused resources. Similar to the KLIK PROM portal, the University of Leeds also does not have data on the PROM completion rate by using PROM education materials.

### Recommendations

While filling out PROMs can be challenging for patients with low health literacy or those with limited education and from diverse and underrepresented communities, we believe that these are not the only hurdles to patient engagement with PROMs completion in routine health care. Education about PROMs should be universally applicable to all patients. To address this, here are few questions for the research and clinical community to consider: (1) what are the best practices in educating or training patients to understand the value and use of PROMs? (2) should there be a better standard for reporting patient education practices in published studies? (3) should there be a standard checklist on the education requirements that need to be reported? (4) should educational activities be evaluated to measure their impact? and (5) should we define and standardize certain terminologies for patient education?

## Conclusion

Our symposium at the 2023 ISOQOL conference brought together a range of experiences and learning around patient-centered PROMs educational activities from Netherlands, Canada, and UK. The symposium also highlighted the gaps and paucity of evidence in the field. While our team will continue working on these issues across our own projects, efforts from the scientific community will be needed to further develop best practices that are inclusive to all patients. Researchers and teams wanting to use PROMs in practice would benefit from better information exchange on best practices for developing and delivering training to patients on PROMs.

## Data Availability

None as this is a commentary.

## Declarations

### Previous presentations of the work

As a symposium with the 2023 ISOQOL Annual Symposium.
